# Relationship Between Bioactive Compounds and Biological Activities (Antioxidant, Antimicrobial, Antihaemolytic) of ‘Colcas’ Fruits at Different Stages of Maturity

**DOI:** 10.3390/antiox14091105

**Published:** 2025-09-10

**Authors:** Elena Coyago-Cruz, Johana Zúñiga-Miranda, Gabriela Méndez, Aida Guachamin, Ruth Escobar-Quiñonez, Carlos Barba-Ostria, Jorge Heredia-Moya

**Affiliations:** 1 Carrera de Ingeniería en Biotecnología, Universidad Politécnica Salesiana, Sede Quito, Campus El Girón, Av. 12 de Octubre N2422 y Wilson, Quito 170143, Ecuador; gmendez@ups.edu.ec (G.M.);; 2 Centro de Investigación Biomédica (CENBIO), Facultad de Ciencias de la Salud Eugenio Espejo, Universidad UTE, Quito 170527, Ecuador; johana.zuniga@ute.edu.ec (J.Z.-M.); jorgeh.heredia@ute.edu.ec (J.H.-M.); 3 Escuela de Medicina, Colegio de Ciencias de la Salud Quito, Universidad San Francisco de Quito (USFQ), Quito 170901, Ecuador; 4 Instituto de Microbiología, Universidad San Francisco de Quito (USFQ), Quito 170901, Ecuador

**Keywords:** in vitro activity, functional foods, *Miconia crocea*, microextractions, maturity degree, medicinal potential

## Abstract

The genus *Miconia* is used in traditional medicine, but there are few studies supporting the bioactive potential of *Miconia crocea*. This study aimed to evaluate the physicochemical properties, bioactive compound content, and antioxidant, antimicrobial and antihaemolytic activities at four different phenological stages of *M. crocea*. The pH, soluble solids, titratable acidity, moisture and ash content were determined. Mineral contents were determined by atomic absorption. Vitamin C, organic acids, carotenoids, chlorophylls and derivatives and phenols were determined by chromatography. Total anthocyanins were determined by spectrophotometry. The antioxidant capacity was evaluated using ABTS and DPPH assays, and the antimicrobial activity was tested against *Escherichia coli*, *Pseudomonas aeruginosa*, *Staphylococcus aureus*, *Streptococcus mutans*, *Candida albicans* and *Candida tropicalis*. Potassium was the predominant mineral (>1000 mg/100 g DW), while malic acid was the predominant organic acid. Lutein was the most abundant carotenoid, as among the phenolic compounds, *m*-coumaric acid and chlorogenic acid were most abundant (>1000 mg/100 g DW). The optimal method for preparing the extract for antimicrobial and haemolytic activity, with a focus on phenols, involved using 50% ethanol, applying ultrasound for six minutes, and carrying out three extractions. The M0% extract exhibited the most potent antimicrobial activity against *S. mutans* (MIC: 7.8 mg/mL). Anti-haemolytic activity indicates biocompatibility. The results emphasise the bioactive and antimicrobial potential of *M. crocea*, suggesting its possible application in various industries. However, further research is needed in the form of in vivo studies.

## 1. Introduction

Ecuador is distinguished by its remarkable wealth of medicinal plants, which indigenous and peasant communities have used for generations to manage diseases. This invaluable ethnobotanical knowledge, passed down from one generation to the next, represents a rich source of new therapeutic alternatives. However, if it is not documented correctly, this knowledge is at risk of being lost. In addition to its exceptional biodiversity, Ecuador’s cultural diversity has fostered the traditional use of numerous wild and cultivated species for medicinal purposes [1].

Fruits and other underutilised plant parts are a potential source of bioactive compounds with functional properties. Phenolic compounds, in particular, are notable for their antioxidant capacity, which contributes to the prevention of chronic non-communicable diseases, including cancer, cardiovascular disease and neurodegenerative disorders. These secondary metabolites also play a crucial role as antimicrobial agents by interfering with the integrity of microbial cell membranes, inhibiting essential enzymes and affecting DNA replication [2,3].

In addition to being responsible for the colour of fruits and flowers, carotenoids act as antioxidants and precursors of vitamin A, and together with essential vitamins such as C and E, they play a key role in modulating the immune system and maintaining cell integrity [4]. Likewise, anthocyanins, which are present in intensely coloured fruits such as grapes and berries, have been associated with reduced oxidative stress and inflammation, as well as improved cardiovascular health [5]. The synergy between these bioactive metabolites gives plant extracts comprehensive functional properties, including antioxidant, antimicrobial and antihaemolytic activity [6,7].

In this context, *Miconia crocea* (Desr.) Naudin is a species belonging to the genus *Miconia.* This genus is the largest taxonomic group within the Melastomataceae family, comprising over 1900 species distributed throughout the Neotropics [8]. Various species of *Miconia* have traditionally been used in folk medicine to treat conditions such as pain, throat infections, fever and colds, as well as to purify, diuretise and sedate. Recent studies have confirmed that these species exhibit various pharmacological activities, including anti-inflammatory, analgesic, antimutagenic, antiparasitic, antimicrobial, antioxidant and cytotoxic properties [9,10]. For example, the leaves of *Miconia albicans* are sold as medicinal infusions, demonstrating their importance in both local medicinal and economic markets [11]. *M. crocea*, commonly known as ‘Colcas’ in Spanish, has been documented in Colombia and Ecuador, although its medicinal potential remains mainly unexplored [12,13]. The fruits are used in specific communities in the Ecuadorian Andes to prepare colada morada, a drink offered on the Day of the Dead. Thus, the present study aimed to evaluate the physicochemical properties, bioactive compound content, antioxidant, antimicrobial and haemolytic activities of *Miconia crocea* at different phenological stages. The results will therefore provide a foundation for future research aimed at generating scientific evidence on the medicinal and functional potential of this underutilised species.

## 2. Materials and Methods

### 2.1. Reagents and Standards

This study employed analytical-grade reagents, high-performance liquid chromatography (HPLC) and standards. Acetone (CAS 67-64-1), fluconazole (CAS8638673-4) and trichloromethane (CAS 67-66-3) were purchased from Fisher Chemical (Fisher Scientific Inc., Madrid, Spain) in analytical grade, while acetonitrile (CAS 75-05-8), ethanol (CAS 64-17-5), ethyl acetate (CAS 141-78-6) and methanol (CAS 67-56-1) were purchased in HPLC grade. ABTS (2,2′-azino-bis-(3-ethylbenzothiazoline-6-sulfonic acid) (CAS 30931-67-0), *DL*-homocysteine (CAS 454-29-5), DPPH (2,2-diphenyl-1-picrylhydrazyl) (CAS 1898-66-4), formic acid (CAS 64-18-6), Folin–Ciocalteu reagent (CAS7732-18-5), metaphosphoric acid (CAS 37267-86-0), nitric acid (CAS7697-37-2), potassium chloride (CAS7447-407), n-acetyl-n,n,n-trimethylammonium bromide (CAS 57-09-0), potassium persulfate (CAS 7727-21-1), potassium monobasic phosphate (CAS 7778-77-0), sodium acetate trihydrate (CAS 6131-90.4), sodium carbonate (CAS 497-19-8), sodium hydroxide (CAS1310-73-2) and sulfuric acid (CAS 7664-93-9), all of which were of analytical grade, were from Sigma-Aldrich (Merck, Darmstadt, Germany). Analytical-grade hydrochloric acid (CAS 7647-01-0) was purchased from Labscan (RCI Labscan Group, Dublin, Ireland). All procedures were carried out using water that had been purified with a NANOpure Diamond™ system (Barnstead Inc., Dubuque, IA, USA).

Various microbiological growth media, including Brain Heart Infusion (BHI), Mueller-Hinton agar (MHA) and Sabouraud dextrose agar (SDA), were obtained from BD DifcoTM (Fisher Scientific Inc., Madrid, Spain). Additionally, Yeast Peptone Dextrose Broth (YPDB) was sourced from SRL (Sisco Research Laboratories Pvt. Ltd., Mumbai, India), while streptomycin sulphate (CAS 3810-74-0) was obtained from Phytotech (PhytoTechnology Laboratories^®^, Lenexa, KS, USA). The water used throughout the study was purified using a NANOpureDiamondTM system (Barnsted Inc., Dubuque, IA, USA).

Standard compounds of a high degree of purity were also used, all of which were provided by Sigma (Merck, Darmstadt, Germany): *L*-(+)-ascorbic acid 99.8% (CAS 50-81-7); citric acid 100.8% (CAS 77-92-9); malic acid 99.0% (CAS 97-67-6); *L*-(+)-tartaric acid 99.5% (CAS 87-69-4); caffeic acid 98.0% (CAS 331-39-5); chlorogenic acid 95.0% (CAS 327-97-9); and chrysin 97.% (CAS 480-40-0), *p*-, *m*- and *o*-coumaric acids (CAS 501-98-4, 588-30-7 and 614-60-8, respectively), cyanidin-3-glucoside chloride 97% (CAS 7084-24-4), 2,5.dihydroxybenzoic acid 98% (CAS 149-91-7), ferulic acid 100% (CAS 1135-24-6), gallic acid 100% (CAS 149-91-7), *p*-hydroxybenzoic acid (CAS 99-96-7), 3-hydroxybenzoic acid 99% (CAS 99-06-3) and 2,5-dihydroxybenzoic acid 98% (CAS 490-79-9), *p*-hydroxybenzoic acid 99% (CAS 99-06-3), kaempferol 97.0% (CAS 520-18-3), luteolin 98.0% (CAS 491-70-3), naringin 95.0% (CAS 10236-47-7), quercetin 95.0% (CAS 84906-19-8), rutin 94.0% (CAS 153-18-4), shikimic acid 99.0% (CAS 138-59-0) and syringic acid 95% (CAS 530-57-4), vanillic acid 97.0% (CAS 121-34-6), β-carotene 93.0% (CAS 7235-40-7), β-cryptoxanthin 97.0% (CAS 472-70-8), lutein (CAS 127-40-2), lycopene (CAS 502-65-8), zeaxanthin (CAS 144-68-3), chlorophyll a (CAS 479-61-8), chlorophyll b (CAS 519-62-0) and Trolox 98.0% (CAS 53188-07-1). In turn, calcium (CAS7440-70-2), iron (CAS 7439-89-6), magnesium (CAS7439-95-4), potassium (CAS7440-09-7) and sodium (CAS7440-23-5) with a concentration of 1000 μg/mL were purchased from Accustandard (AccuStandard, Inc., New Haven, CT, USA). The microbial strains employed in this study included *Candida albicans* ATCC 1031, *Candida tropicalis* ATCC 13803, *Escherichia coli* ATCC 8739, *Pseudomonas aeruginosa* ATCC 9027, *Staphylococcus aureus* ATCC 6538P and *Streptococcus mutans* ATCC 25175, all of which were procured from the American Type Culture Collection (ATCC), based in Manassas, VA, USA.

### 2.2. Material Studied

**Figure 1:**
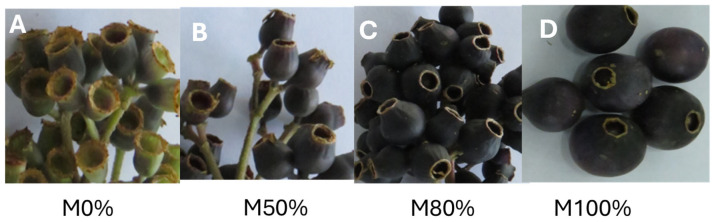
*Miconia crocea* (‘Colcas’) at different phenological stages of development. Note: (**A**), Fruit set (7 weeks after pollination); (**B**), 50% fruit development (11 weeks after pollination); (**C**), 80% fruit development (13 weeks after pollination); (**D**), total maturity (16 weeks after pollination).

The study considered ‘Colcas’ fruits (*Miconia crocea* (Desr.) Naudin.) at various stages of phenological development, as determined by the number of weeks since pollination (Figure 1). Thus, the M0% stage corresponded to fruit that had undergone 7 weeks of phenological development; the M50% stage, 11 weeks; the M80% stage, 13 weeks; and the M100% stage corresponded to mature fruit that had undergone 16 weeks of phenological development. Samples were collected in the province of Tungurahua in Ecuador (geographical coordinates: 1°22′3.4″ S, 78°34′8.6″ W). To confirm the taxonomic identity of the species, botanical samples were collected and subsequently authenticated in the herbarium of the Universidad Politécnica Salesiana (Identification code: 0004527, Herbario QUPS-Ecuador).

### 2.3. Physicochemical Analyses

Samples were randomly selected from the same sector, with a sample size of 2 kg considered for the respective analyses. The sample was divided into two portions. The first portion of fresh fruit was evaluated for pH, soluble solids (°Brix), total titratable acidity by titration, moisture at 110 °C in an Memmert Be 20 oven (Memmert GmbH + Co. KG, Barcelona, Spain) and ash at 550 °C in a muffle furnace (Thermo Fisher Scientific, Waltham, MA, USA). The second portion was frozen at −80 °C and freeze-dried using a Christ Alpha 1–4 LDplus (GmbH, Osterode am Harz, Germany). The dried samples were crushed and stored in amber vials under a nitrogen atmosphere until the respective analysis [3].

#### Mineral Profile

To extract the minerals, 40 mg of freeze-dried powder was weighed into a Speedwave Xpert microwave digestion vessel (Berghof Products + Instruments GmbH, Eningen unter Achalm, Germany) and mixed with 5 mL of 65% nitric acid. The mixture was left to stand for 10 min before the digester was closed. Digestion was then carried out using a linear gradient: 140 °C, 30 bar and 70% power for 5 min; 200 °C, 35 bar and 80% power for 15 min; and finally, 50 °C, 25 bar and 0% power for a further 10 min. After digestion, the vessels were left to cool to room temperature for 20 min, and the sample was transferred to a 25 mL flask. This was washed with Milli-Q water, weighed and stored in an amber glass bottle until analysis [14].

Mineral quantification was performed using a Varian SpectrAA-55 atomic absorption spectrophotometer (Agilent Technologies, Mississauga, ON, Canada). Calcium was determined at a wavelength of 422.7 nm with a slit width of 0.5 nm and using a mixture of acetylene and nitrous oxide. Iron, magnesium, potassium and sodium were analysed using a mixture of acetylene and air. These were measured at wavelengths of 372.0 nm, 202.6 nm and 589.6 nm, respectively, with slit widths of 0.2 nm, 1.0 nm, and 0.5 nm, respectively. The mineral standards were based on a 1000 ppm standard solution, and the following concentrations were prepared at 0–5 ppm for Ca, 0–20 ppm for Fe, 0–200 ppm for K, 0–10 ppm for Mg and 0–8 ppm for Na. The analysis was carried out in triplicate. Concentrations were expressed in milligrams of mineral per 100 g of dry weight (mg/100 g DW) [15].

### 2.4. Bioactive Compounds Content

#### 2.4.1. Ascorbic Acid

To determine the *L*-ascorbic acid (vitamin C) content, 40 mg of the freeze-dried powder sample was mixed with 1.2 mL of a 3% metaphosphoric acid solution and 0.2 mL of a 0.2% *DL*-homocysteine solution. This was then homogenised using a VM-300 vortex mixer (Interbiolab Inc., Orlando, FL, USA) before being ultrasonically stirred for three minutes in an FS60 bath sonicator (Fisher Scientific Inc., Waltham, MA, USA). The final volume of the extract was adjusted to 2 mL with Milli-Q ultrapure water. The solution was then filtered with a 0.45 µm PDVF filter and injected into a Model 1200 RRLC Liquid Chromatography system (Agilent Technologies, Mississauga, ON, Canada) equipped with a Diode Array Detector (DAD-UV–VIS) at 244 nm and a ZORBAX Eclipse XDB 80 Å C18 Column (1.8 μm, 4.6 mm × 50 mm) (Agilent Scientific Instruments, Santa Clara, CA, USA). A calibration curve was obtained using different injection volumes (2–20 μL) of a 0.1 mg/mL standard solution of ascorbic acid. The analysis was carried out in triplicate. The results were expressed as milligrams of ascorbic acid per 100 g of dry weight (mg/100 g DW) [3]. All analyses were performed in triplicate.

#### 2.4.2. Organic Acid Profile

To extract the organic acids, 40 mg of the lyophilised sample was weighed and mixed with 1.5 mL of a solution containing 0.02 N sulfuric acid, 0.05% metaphosphoric acid and 0.02% homocysteine. The mixture was then homogenised and ultrasonically shaken for three minutes. This was then filtered through a 0.45 µm PDVF filter, after which the resulting solution was quantified using a 1200 RRLC liquid chromatograph (Agilent Technologies, Mississauga, ON, Canada) equipped with a DAD-UV–VIS detector at 210 nm and a YMC-Triart C18 column (3 µm, 4.6 mm × 150 mm) (YMC Europe GmbH, Dinslaken, Germany). A calibration curve was constructed using 100 mg/mL standards of citric, malic and tartaric acids, spiked at different volumes (between 2 and 20 µL) and analysed separately. The analysis was carried out in triplicate. The results were expressed as milligrams of organic acid per 100 g of dry weight (mg/100 g DW) [3]. All analyses were performed in triplicate.

#### 2.4.3. Carotenoid Profile

For the extraction of carotenoids, 20 mg of the lyophilised sample was mixed with a solution of methanol, acetone and dichloromethane in a ratio of 1:1:2. The solution was then homogenised and ultrasonicated for 2 min. This procedure was repeated successively until complete extraction of the coloured compounds present in the solid was achieved. The coloured phase was recovered and evaporated under reduced pressure using a rotary evaporator at a temperature below 30 °C [15].

Quantification followed the methodology of Coyago et al. [3]. The dry extract was rediluted in 20 μL of ethyl acetate and analysed on a 1200 RRLC liquid chromatograph (Agilent Technologies, Mississauga, ON, Canada), equipped with a DAD-UV–VIS detector and a YMC C30 column (3 μm, 4.6 × 150 mm) (YMC Europe GmbH, Dinslaken, Germany). Identification and quantification were performed using reference standards for astaxanthin, α-carotene, β-carotene, β-cryptoxanthin, lycopene, lutein, trans-β-apo-8-carotenal, violaxanthin and zeaxanthin. The analysis was carried out in triplicate. The total carotenoid content was expressed as the sum of the individual compounds, reported in milligrams per 100 g of dry weight (mg/100 g DW).

#### 2.4.4. Chlorophylls and Their Derivatives

Chlorophyll and its derivatives were extracted and quantified using the method described in Section 2.4.3. A calibration curve was constructed using 2–20 µL injections of standard solutions of chlorophyll a (0.1 mg/mL), pheophytin a and pheophytin b, with the concentration expressed in milligrams per 100 g of dry weight.

#### 2.4.5. Total Anthocyanins

Quantification was performed using the methodology of Mayorga-Ramos et al. [16]. To extract the anthocyanins, 40 mg of the freeze-dried sample was weighed and then mixed with 2 mL of 96% ethanol. The mixture was then homogenised and shaken in an ultrasonicator for three minutes. The resulting supernatant was recovered by centrifugation at 1400 rpm for 5 min at 4 °C, after which it was stored at −80 °C until analysis.

Anthocyanin quantification used two 50 µL aliquots of the supernatant, which were placed in a 96-well VWT microplate (Corning, Glendale, AZ, USA). The first was mixed with 200 µL of a 0.025 M potassium chloride buffer solution (pH 1), and the second with 200 µL of a 0.4 M sodium acetate buffer solution (pH 4.5). The absorbencies of both solutions were recorded at 520 nm and 700 nm using a spectrophotometer equipped with a microplate reader. Quantification was performed using a calibration curve constructed with cyanidin-3-glucoside in the range of 0.05 to 0.2 mg/mL. The analysis was carried out in triplicate. The results were expressed as milligrams of cyanidin-3-glucoside chloride per 100 g of dry weight (mg C-3-gl/100 g DW). All analyses were performed in triplicate.

#### 2.4.6. Phenol Profile

Total phenol extraction and quantification were performed according to the methodology of Coyago et al. [17]. Phenols from 20 mg of lyophilised powder were extracted with 100 µL of an 80% methanol solution acidified with 0.1% HCl. The mixture was homogenised and ultrasonicated for three minutes; the supernatant was recovered, and the solid was extracted several times with 500 µL of the solution. The mixture of the supernatants was stored until the respective analysis was performed. Total phenol quantification was performed using the Folin–Ciocalteu method with gallic acid as a standard. This was carried out using 96-well microplates by mixing the extracts or standards with the Folin–Ciocalteu reagent and sodium carbonate. The reaction was then allowed to proceed in the dark for two hours. Absorbance was then measured at 750 nm. The results were expressed as milligram equivalents of gallic acid per 100 g of dry weight (mg EAG/100 g DW).

Individual phenol quantification was performed using the methodology described by Zuñiga-Miranda et al. [18]. To identify individual phenolic compounds, the extracts were filtered and analysed by RRLC-DAD 1200 using a Zorbax Eclipse Plus C18 column (4.6 × 150 mm, 5 μm) (Agilent Technologies, Santa Clara, CA, USA), a binary gradient system, and a mixture of formic acid and acetonitrile. Selecting wavelengths of 280, 320 and 370 nm allowed various compounds to be identified by comparing their UV–Vis spectra and retention times with those of reference standards. Standards such as gallic acid, caffeic acid, ferulic acid, quercetin and rutin were used. The analysis was carried out in triplicate. The concentration of each compound was reported in milligrams per 100 g of dry weight. All analyses were performed in duplicate or triplicate to ensure data accuracy and reliability.

### 2.5. Antioxidant Activity Analyses

The antioxidant capacity was evaluated using the ABTS and DPPH methods, following the methodology of Mayorga-Ramos et al. [16]. The analysis was carried out in triplicate. The extract was prepared by mixing 20 mg of the lyophilised sample with 2 mL of methanol. The mixture was homogenised, stirred in an FS60 ultrasonic bath (Fisher Scientific Inc., Waltham, MA, USA) for 3 min, and the supernatant was recovered by centrifugation at 4 °C, 5 min, and 14,000 rpm.

The ABTS^•+^ radical was generated by mixing equal volumes of 2.45 mM potassium persulfate and 7 mM ABTS. This mixture was then left to stand for 16 h before being diluted with ethanol until an absorbance of 0.70 ± 0.02 at 754 nm was achieved. Quantification was performed by placing 10 µL of the standard or extract into a 96-well plate (VWR, Novachen, Pittsburgh, PA, USA) along with 200 µL of the ABTS^•+^ radical. Blanks were also included for methanol, the ABTS^•+^ radical and the extract. Absorbance was measured at 754 nm in a BioTek microplate spectrophotometer (Agilent Scientific Instruments, Santa Clara, CA, USA). ABTS antioxidant activity was quantified using a 10 mM Trolox standard diluted between 0.2 and 0.7 mM, with R^2^ greater than 0.99. Antioxidant capacity was expressed in millimoles of Trolox equivalents per 100 g of dry weight.

The DPPH^•^ radical was generated by mixing 10 mg of DPPH with 50 mL of HPLC methanol. For quantification, 20 µL of the standard or extract was mixed with 280 µL of DPPH radical in a 96-well plate. Methanol, the DPPH^•^ radical and extract blanks were also used. The plate was then incubated in the dark on a 4310-plate shaker (Fisher Scientific, USA) for 30 min. Absorbance was measured at 560 nm using a BioTek microplate spectrophotometer (Agilent Technologies, Santa Clara, California, USA). DPPH antioxidant activity was quantified using a 10 mM Trolox standard diluted between 0.4 and 4 mM, with R^2^ greater than 0.99. Antioxidant capacity was expressed in millimoles of Trolox equivalents per 100 g of dry weight.

### 2.6. Antimicrobial Activity Analyses

The aim was to obtain an extract with a high concentration of phenolic compounds using a green extraction approach. To this end, a 3 × 3 factorial experimental design was employed. This design incorporated three variables: solvent type (100% ethanol, 50% ethanol, and 100% water), sonication time (1, 6, and 10 min) and number of extractions (1, 2, and 3).

Due to the limited availability of freeze-dried ‘Colcas’ fruits and other species in the same genus, the extraction was validated using a pulp with similar colour and texture characteristics, such as that from *Solanum betaceum* variety ‘Morado gigante’. For each treatment, 1500 mg of lyophilised sample was weighed, then 250 mL of the corresponding solvent was added. The mixture was homogenised and subjected to ultrasound for the times specified in the experimental design. The supernatant was then recovered by centrifugation at 7500 rpm for 5 min at 4 °C using a Model 5430R centrifuge (Eppendorf AG, Hamburg, Germany).

All extractions were performed according to the defined factorial design protocol, and the obtained supernatants were quantified by total phenol analysis, following the methodology described in Section 2.4.6. The treatment with the highest concentration of phenolic compounds was selected for further analysis of its antimicrobial and haemolytic activity.

#### 2.6.1. Antibacterial Activity

The antibacterial activity of ‘Colcas’ at different phenological stages was determined using the antibiogram technique for initial screening. The bacterial inoculum was prepared in brain–heart infusion broth (BHI) and adjusted to a 0.5 MacFarland standard. This suspension was spread uniformly on solidified Muller−Hinton Agar (MHA) using a sterile swab. A fixed volume of 80 μL, containing a concentration of the fruit extract of 300 mg/mL, was added to the wells. The Petri plates were then incubated at 37 °C for 24 h. Streptomycin sulphate salt (1400 µg/mL) served as the positive control. Additionally, water was used as a control. The analysis was carried out in triplicate.

##### Minimal Inhibitory Concentration (MIC)

‘Colcas’ at M0% and M100% was used to determine the MIC, because they represent the physiological and chemical extremes of the ‘Colcas’ fruit. The MIC was evaluated according to the Clinical and Laboratory Standards Institute (CSLI) guidelines by comparing the optical density (OD600) at time 0 with the value after 24 h [19,20]. The bacterial inoculum was prepared in brain–heart infusion broth (BHI) and adjusted to a 0.5 MacFarland standard. Stock solutions of the extracts were prepared by resuspending them in water at 300 mg/mL. This was diluted in series eight times in water. Then, 100 µL of each dilution and 100 µL of the bacterial suspension were added to a sterile 96-well microplate. Additionally, Streptomycin sulphate salt (1400 µg/mL) was used as a control for growth inhibition at the recommended working concentrations for the tested strains. Both BHI alone and supplemented with the extracts at different concentrations were used as blanks. The plates were then incubated at 37 °C for 24 h. These assays were performed at least in triplicate.

#### 2.6.2. Antifungal Activity

The antifungal activity of ‘Colcas’ fruit extracts was determined using the antibiogram technique for initial screening. The fungal inoculum was prepared in Yeast Peptone Dextrose Agar (YPD) and adjusted to a 0.5 MacFarland standard. This suspension was spread uniformly on solidified Agar YPD using a sterile swab. A fixed volume of 80 μL, containing a concentration of the fruit extract of 300 mg/mL, was added to the wells. The Petri plates were then incubated at 37 °C for 48 h. Fluconazole (1500 µg/mL) served as the positive control. Additionally, water was used as a control. The analysis was carried out in triplicate.

##### Minimal Inhibitory Concentration (MIC)

Fruit pulp extracts M0% and M100% that present an inhibition zone for the well diffusion assay were used to determine the MIC. The MIC was evaluated according to the Clinical and Laboratory Standards Institute (CSLI) guidelines [19,20,21]. The fungal inoculum was prepared in YPD to a final cell density of 1.5 × 10^6^ CFU/mL. Stock solutions of the fruit pulp extracts were prepared by resuspending them in water at a concentration of 300 mg/mL. This was diluted in series eight times in water. Then, 100 µL of each dilution and 100 µL of the bacterial suspension were added to a sterile 96-well microplate. Additionally, fluconazole (1500 μg/mL) was used as a control for growth inhibition at the recommended working concentrations for the tested strains. Both YPD alone and supplemented with the extracts at different concentrations were used as blanks. The plates were then incubated at 37 °C for 24 h. These assays were performed at least in triplicate.

### 2.7. Haemolytic Activity

The haemolytic potential of ‘Colcas’ fruit extracts was assessed through a modified spectrophotometric assay, adapted from established protocols to enhance sensitivity and analytical robustness [22]. Defibrinated sheep blood (10 mL) was washed three times with phosphate-buffered saline (PBS 1×, pH 7.4) by centrifugation (1700× *g*, 5 min), and the erythrocytes were re-suspended to yield a 1% suspension in PBS 1×. This suspension was mixed in equal volumes (1:1) with either the ‘Colcas’ fruit extract at indicated concentrations (2500, 1250, 625, 312.5 and 156.25 μg/mL), a positive control (10% Triton X-100 to induce haemolysis) or a negative control (PBS 1×). Each mixture was incubated in polypropylene 96-well plates for 1 h at 37 °C under gentle agitation. After incubation, samples were centrifuged (1700× *g*, 5 min), and the supernatants were carefully transferred to flat-bottom, transparent 96-well plates for spectrophotometric analysis.

To capture the haemoglobin release with maximal spectral resolution, absorbance spectra were acquired from 340 nm to 800 nm (10 nm intervals) using a Cytation5 multi-mode plate reader (BioTek Instruments, Winooski, Vermont, USA). Colour controls—extract dilutions in PBS without erythrocytes—were included in parallel to correct for background absorbance arising from the intrinsic pigmentation of the extract. This step was critical to avoid overestimation of haemolysis due to spectral interference.

Each condition was analysed in triplicate, and the experiment was repeated independently on three separate occasions to ensure reproducibility. For quantification, a weighted absorbance value (OD_pond_) was calculated based on the characteristic absorbance maxima of oxyhaemoglobin at 410, 540 and 580 nm. Weights of 0.6, 0.2 and 0.2 were respectively applied to emphasise the dominant Soret band at 410 nm while accounting for contributions from the α and β bands, thereby minimising the influence of random fluctuations and non-specific interference:

OD_pond_ = 0.6 × OD_410_ + 0.2 × OD_540_ + 0.2 × OD_580_

The percentage haemolysis rate (%HR) was subsequently calculated using the normalised formula:

%HR = (OD_sample pond_ − Od_neg pond_)/(OD_pos pond_ − O_dneg pond_) × 100

### 2.8. Statistical Analysis

Statistical analyses were performed using STATGRAPHICS Centurion XVII, RStudio (version 4.4.1), and SigmaPlot (version 14.0). Results are expressed as mean ± standard deviation (SD). A one-way analysis of variance (ANOVA) was conducted, and pairwise comparisons between group means were performed using Tukey’s honest significant difference (HSD) test at a significance level of *p* < 0.05. Pearson correlation coefficients were calculated at a 95% confidence level to assess the relationships among different stages of physiological development. To identify the variables most strongly associated with differentiation among maturity stages, a principal component analysis (PCA) was carried out. All measurements were performed in triplicate.

The PCA included the complete set of evaluated variables, such as mineral content, vitamin C, carotenoids, total anthocyanins, total phenolics, organic acids, antioxidant capacity (measured via ABTS and DPPH assays) and antimicrobial activity (expressed as inhibition zone diameter in millimetres). Given the heterogeneity in measurement units and scales, data were standardised by centring (subtracting the mean) and scaling to unit variance (dividing by the standard deviation), ensuring all variables had a mean of 0 and a variance of 1. This standardisation prevents bias toward variables with larger magnitudes and ensures equal contribution of all parameters to the multivariate analysis. Although all possible principal components were computed, the first two components accounting for 79.2% of the total variance were selected for visualisation and clustering analysis due to their high cumulative explained variance.

## 3. Results

### 3.1. Physicochemical Characteristics

The physical and chemical characteristics of a fruit are essential factors in terms of acceptability. Table 1 shows the average values for the weight, size, pH level, soluble solids content, titratable acidity, moisture content and ash content of ‘Colcas’ at various stages of phenological development. It also presents the mineral profile, including calcium, iron, potassium, magnesium and sodium.

### 3.2. Analysis of Bioactive Compounds

Bioactive compounds are molecules that contribute to human health when ingested as part of the diet. Table 2 shows the concentrations of vitamin C and the profiles of organic acids, carotenoids, chlorophylls and their derivatives, and phenolic compounds at the different stages of ‘Colcas’ phenological development.

### 3.3. Antioxidant Activity Analyses

Antioxidants contribute to the neutralisation of free radicals and the prevention of diseases related to oxidative stress. Table 3 shows the average antioxidant activity values evaluated using the DPPH and ABTS methods at the different stages of ‘Colcas’ development.

### 3.4. Antimicrobial Activity Analyses

**Figure 2:**
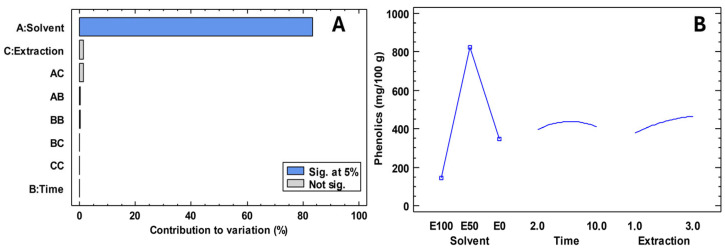
Results of the experimental design for the extraction of compounds for antimicrobial activity. Note: E100; ethanol 100%; E50; ethanol 50%; E0, water 100%. (**A**) Contribution to variation; (**B**) Experimental design.

The misuse of medicines has led to disease-causing microorganisms becoming a public health problem. In this regard, it has become necessary to find new natural alternatives to mitigate the problem. The extraction of compounds that contribute to antimicrobial activity is not well understood, which is why Figure 2 presents an experimental design focused on maximising phenolic compounds. Table 4 shows the antimicrobial activity of *Escherichia coli*, Pseudomonas aeruginosa, Staphylococcus aureus, Streptococcus mutans, Candida albicans and Candida tropicalis in freeze-dried extracts of ‘Colcas’ at various stages of phenological development. Furthermore, Table 5 shows the minimum inhibitory concentration.

### 3.5. Haemolytic Activity

The haemolytic activity of ‘Colcas’ fruit extract was evaluated through spectrophotometric quantification of haemoglobin release following erythrocyte exposure. Positive controls treated with 10% Triton X-100 yielded characteristic oxyhaemoglobin absorbance peaks at 410 nm (2.50 ± 0.15), 540 nm (0.309 ± 0.025) and 580 nm (0.153 ± 0.018), resulting in a weighted optical density (OD_pon_d) of 1.59 ± 0.12. Negative controls (PBS 1×), by contrast, showed baseline absorbance (OD_pon_d = 0.052 ± 0.008), confirming negligible spontaneous haemolysis under the assay conditions.

After correcting for the intrinsic colour of the extract by subtracting absorbance values from the corresponding colour controls, a clear trend was observed across the concentration series. The %HR values suggest that, while there is a marginal increase in absorption at the highest concentration, it remains substantially below the positive control reference.

Corresponding haemolysis rates (%HR), calculated using the normalised equation shown in Section 2.6, were minimal across all tested concentrations. At 2500 μg/mL, the extract produced a mean %HR of approximately 3%, with even lower values recorded at 1250 μg/mL and below, falling below 2% and approaching background levels at concentrations of 625 μg/mL, 312.5 μg/mL and 156.25 μg/mL.

**Figure 3:**
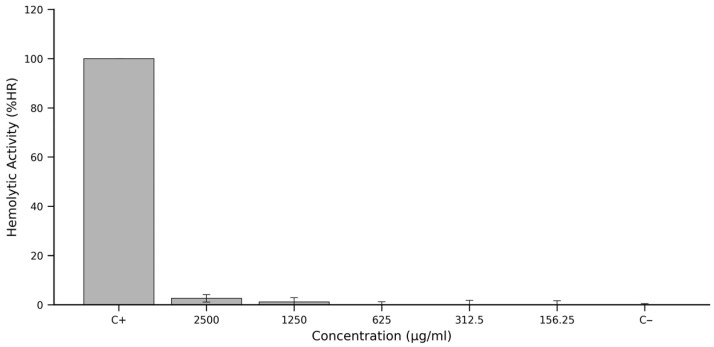
Haemolytic activity (%HR) of *Miconia crocea* (‘Colcas’ fruit) extract across a concentration gradient. Note: The percentage of haemolysis was quantified by measuring oxyhaemoglobin release from sheep erythrocytes. Triton X-100 (10%) served as the positive control (C+) for 100% haemolysis, and PBS 1× as the negative control (C−). Data represent the mean ± standard deviation from three independent experiments, each with three technical replicates. The extract exhibited minimal haemolytic activity at all concentrations tested, remaining below 3% even at the highest dose, indicating a favourable hemocompatibility profile.

These findings, graphically summarised in Figure 3, underscore the hemocompatibility of the ‘Colcas’ fruit extract. Even at concentrations as high as 2500 μg/mL, haemolytic activity remains negligible, suggesting the absence of membrane-disruptive constituents. The extract thus satisfies one of the initial benchmarks for biocompatibility and supports its continued investigation for safe biological or therapeutic use.

### 3.6. Statistical Analysis

**Figure 4:**
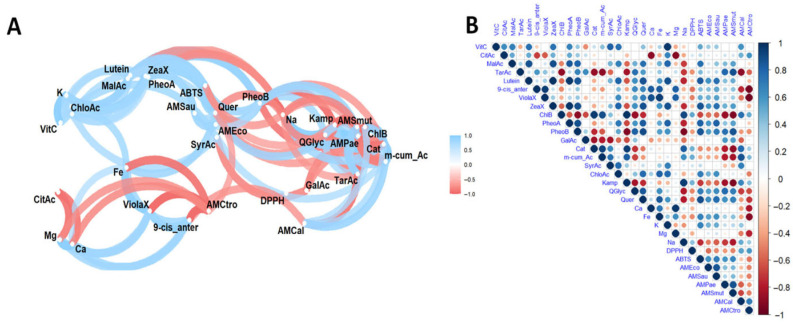
Pearson correlation (**A**) and heat map (**B**) analysis of the studied variables. Note: Violaxanthin (ViolaX), 9-cis anterazanthin (0-cis_ante), lutein (Lutein), zeaxanthin (ZeaX), Zeionaxanthin (ZeioX), chlorophyll b (ChlB), pheophytin a (PheoA), pheophytin b (PheoB), chlorophyll a (ChlA), α-carotene (A-Car), β-carotene (B-Car), vitamin C (VitC), citric acid (CitAc), malic acid (MalAc), tartaric acid (TarAc), gallic acid (GalAc), catechin (Cat), *m*-cumaric acid (m_cum_Ac), 4-hydroxybenzoic acid (4-HBAc), syringic acid (SyrAc), chlorogenic acid (ChloAc), caffeic acid (CafAc), ferulic acid (FerAc), rutin (Ruin), kaempferol (Kamp), quercetin glycoside (QGlyc), quercetin (Quer), calcium (Ca), iron (Fe), potassium (K), magnesium (Mg), sodium (Na), antioxidant activity by DPPH (DPPH), antioxidant activity by ABTS (ABTS), antimicrobial activity against *S. aureus* (AMSau), antimicrobial activity against *E. coli* (AMEco), antimicrobial activity against *P. aeruginosa* (AMPae), antimicrobial activity against *S. mutans* (AMmut), antimicrobial activity against *C. albicans* (AMcal), antimicrobial activity against *C. tropicalis* (AMctro).

**Figure 5:**
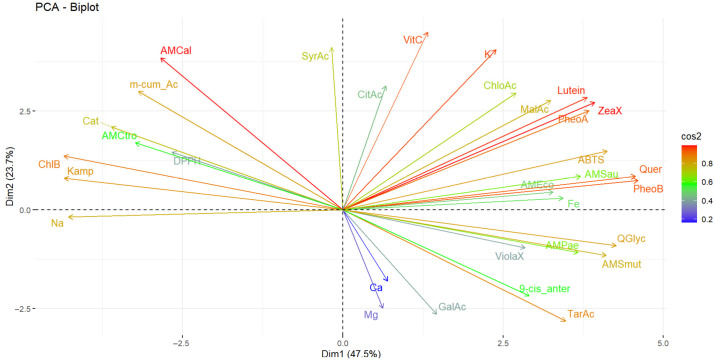
Principal component analysis of the studied variables. Note: Violaxanthin (ViolaX), 9-cis anterazanthin (0-cis_ante), lutein (Lutein), Zeaxanthin (ZeaX), Zeionaxanthin (ZeioX), chlorophyll b (ChlB), pheophytin a (PheoA), pheophytin b (PheoB), chlorophyll a (ChlA), α-carotene (A-Car), β-carotene (B-Car), vitamin C (VitC), citric acid (CitAc), malic acid (MalAc), tartaric acid (TarAc), gallic acid (GalAc), catechin (Cat), *m*-cumaric acid (m_cum_Ac), 4-hydroxybenzoic acid (4-HBAc), syringic acid (SyrAc), chlorogenic acid (ChloAc), caffeic acid (CafAc), Ferulic acid (FerAc), rutin (Ruin), kaempferol (Kamp), quercetin glycoside (QGlyc), quercetin (Quer), calcium (Ca), iron (Fe), potassium (K), magnesium (Mg), sodium (Na), antioxidant activity by DPPH (DPPH), antioxidant activity by ABTS (ABTS), antimicrobial activity against *S. aureus* (AMSau), antimicrobial activity against *E. coli* (AMEco), antimicrobial activity against *P. aeruginosa* (AMPae), antimicrobial activity against *S. mutans* (AMmut), antimicrobial activity against *C. albicans* (AMCal), antimicrobial activity against *C. tropicalis* (AMCtro).

The results of the multivariate analysis performed using networks are presented in Figure 4A, and the heat map in Figure 4B. In turn, Figure 5 presents the principal component analysis of the samples under study.

## 4. Discussion

### 4.1. Physicochemical

The physicochemical parameters of *Miconia crocea* (‘Colcas’) exhibited significant changes across the various stages of its phenological development. The pH decreased progressively from 3.4 in M0% to 2.8 in M100%, indicating gradual acidification of the plant tissue during ripening. This reduction in pH is typically accompanied by an increase in organic acid accumulation, a phenomenon widely documented in both climacteric and non-climacteric fruits [23].

Conversely, total soluble solids (°Brix) increased significantly from 1.2 °Brix at M0% to 6.2 °Brix at M100%, indicating the accumulation of simple sugars such as glucose, fructose and sucrose. This behaviour is typical of ripening fruits, as reported in studies on plums and blackberries, where the increase in soluble solids is linked to the conversion of starches to sugars, thereby improving the fruit’s organoleptic characteristics [24].

Titratable acidity exhibited opposite behaviour, rising from 0.3% at M0% to 1.2% at M100%. This may be due to an increased concentration of organic acids, such as malic and citric acids, which accumulate during the primary and secondary metabolic processes of the fruit during advanced ripening. This result is consistent with previous observations in similar species, where total acidity increases at the end of development as a defence and metabolic conservation mechanism [23,25].

The moisture content increased with maturation, ranging from 81.5% at M0% to 87.2% at M100%. This behaviour may be associated with greater water retention due to cell expansion and softening of plant tissues during the senescence process. Ash content peaked at M80% (1.4%) and was lowest at M100% (0.8%). This could be related to the redistribution and mobilisation of minerals during the final stages of fruit development. This phenomenon has been observed in blackberry varieties, where behaviour varies depending on the specific variety [25].

Mineral analysis revealed that potassium was the predominant element throughout the phenological stages, reaching a maximum value of 3109.5 mg/100 g DW at M80%. This was followed by calcium (maximum value of 1263.9 mg/100 g DW at M0%), magnesium (maximum value of 254.4 mg/100 g DW at M80%) and sodium (maximum value of 24.9 mg/100 g DW at M0%). The high potassium content is characteristic of tropical fruits and is associated with key processes, including osmotic regulation, sugar synthesis and nutrient transport. Calcium concentration was higher in the early stages (M0%), suggesting its structural role in the cell wall, with a progressive decrease resulting from degradation processes during ripening [26]. Iron was only detected in M50% and M80%, with a maximum concentration of 22.0 mg/100 g DW in the latter stage. The absence of detectable iron in M0% and M100% could be due to low bioavailability or transient involvement in specific metabolic processes.

### 4.2. Bioactive Compounds

‘Colcas’ contains bioactive compounds that are an important source of secondary metabolites with potential health benefits for humans. Significant variations in the concentration of *L*-ascorbic acid, anthocyanins, organic acids, carotenoids, chlorophylls and phenolic compounds were observed at different stages of phenological development.

*L*-ascorbic acid is one of the most important water-soluble antioxidants found in plants. It can eliminate free radicals, protect biomolecules and reduce the risk of chronic diseases, including certain types of cancer. Since humans cannot synthesise this vitamin themselves, fruit is an essential dietary source [23]. The *L*-ascorbic acid content exhibited a non-linear pattern, ranging from 0.9 mg/100 g DW (M100%) to 4.1 mg/100 g DW (M50%). This behaviour suggests that the intermediate stage of development is optimal for ascorbic acid biosynthesis, possibly due to increased antioxidant metabolic activity. The decrease in M100% may be related to increased oxidative metabolism and the action of enzymes such as ascorbate oxidase, which has been observed in species including peppers and mandarins [27,28]. Other studies on ripe fruit, however, have reported concentrations of up to 11.2 mg/100 g DW [3]. The variability observed between studies could be attributed to differences in variety, degree of ripeness, harvest time and soil and climate conditions [29].

The organic acid profile showed a high concentration of malic acid, followed by citric and tartaric acid. Malic acid reached its maximum value at M50% (52,803.5 mg/100 g DW), while the highest values for citric and tartaric acids were observed at M50% (1059.8 mg/100 g DW) and M100% (730.6 mg/100 g DW), respectively. The total sum of organic acids was highest at M50% (6706.6 mg/100 g DW), although a high concentration was also observed at M100% (5421.2 mg/100 g DW).

These results suggest that organic acids play a significant role in pH regulation, energy metabolism and the biosynthesis of secondary compounds. The accumulation of tartaric acid in M100% could be associated with catabolic processes related to senescence. The low concentration of *L*-ascorbic acid relative to the high levels of organic acids suggests the conversion of ascorbic acid into compounds such as oxalate, tartrate and threonate, as has been observed in tomato and blackberry fruits [3,24,27].

The carotenoid profile of ‘Colcas’ was dominated by lutein, followed by 9-cis-antheraxanthin, violaxanthin and zeaxanthin. The total carotenoid concentration ranged from 3.1 mg/100 g DW at M0% to 10.0 mg/100 g DW at M50%, subsequently decreasing at M100%. Lutein was the predominant carotenoid, reaching a maximum value of 9.1 mg/100 g DW at M50%. Violaxanthin and zeaxanthin were only detected at M50% and M80%, respectively.

These results demonstrate intense xanthophyll cycle activity during the middle stages of development, potentially in response to light stress and as a means of photoprotective regulation. This process has been observed in other species, such as tomatoes and citrus fruits, where the transition from chloroplasts to chromoplasts during ripening involves reprogramming carotenoid-specific gene expression [30,31]. The reduction observed at M100% may be due to the oxidative degradation of carotenoids or their conversion to volatile apocarotenoids.

The profile of chlorophylls and their derivatives in ‘Colcas’ revealed a distinct metabolic shift throughout the fruit’s developmental stages. Chlorophyll b decreased progressively from 15.0 mg/100 g DW in M0% to 0.1 mg/100 g DW in M100%. In contrast, pheophytin a and b (chlorophyll degradation products) exhibited higher concentrations in M50% (10.3 and 88.7 mg/100 g DW, respectively). The total concentration of chlorophyll pigments and derivatives was highest in M50% (102.0 mg/100 g DW), indicating an active transition phase between photosynthetically active and maturing tissues.

This pattern indicates that, as fruit development progresses, chlorophyll is degraded, generating compounds such as pheophytins that accumulate temporarily before being converted into non-fluorescent catabolites. This process is typical during the senescence or ripening of non-climacteric fruits and has been well documented in species such as tomatoes, peppers and grapes [32]. Chlorophyll degradation is closely regulated by environmental factors (light, pH) and enzymes (chlorophyllase, pheophytinase), and its progression coincides with the visual change of the fruit from green to red or purple, accompanied by an increase in carotenoids and anthocyanins [31,32].

The total amount of anthocyanins increased progressively during development, with values ranging from 10.9 mg/100 g DW (M0%) to 91.3 mg/100 g DW (M100%). This increase is associated with fruit ripening, during which a colour change from green to red occurs because of the accumulation of pigments such as anthocyanins and carotenoids. These molecules provide colour and have potent antioxidant and anti-inflammatory properties [31]. Studies on blackberry varieties have also reported an increase in anthocyanins depending on the degree of ripeness [25].

The total phenolic compound content showed significant variations between phenological stages, with a maximum at M50% (7539.9 mg/100 g DW) and a minimum at M100% (2844.5 mg/100 g DW). Among the most abundant individual compounds were m-coumaric acid and chlorogenic acid, followed by catechin, kaempferol, quercetin and their derivatives.

Phenolic acids showed higher concentrations in the early and intermediate stages (M0% and M80%), indicating their role in antioxidant defence against oxidative stress. For example, chlorogenic acid reached its highest concentration at M80% (4058.8 mg/100 g DW), while *m*-coumaric acid predominated at M0% (3308.1 mg/100 g DW). Flavonoids such as catechin and kaempferol were higher at M0%, while quercetin and its glycoside reached their peak at M80%. This behaviour is in line with previous studies, which have shown that phenolic compounds vary depending on the degree of ripeness, environmental stress, and agronomic conditions [3,33].

The decrease observed in M100% could be due to the enzymatic degradation, oxidation or metabolic utilisation of these compounds during the senescence process. As described in fruits such as Prunus domestica, flavonoids tend to decrease and anthocyanins increase as ripening progresses, thereby changing the fruit’s functional profile [23].

### 4.3. Antioxidant Activity

The antioxidant activity of ‘Colcas’ extracts, as determined by DPPH and ABTS assays, was consistent with the content of phenolic compounds. The DPPH assay revealed an average value of 5.3 mmol TE/100 g DW, whereas the ABTS assay showed values ranging from 5.5 mmol TE/100 g DW at M0% to 6.1 mmol TE/100 g DW at M50% and M80%. This suggests that ‘Colcas’s have a higher antioxidant capacity during the middle phenological stages, when the concentration of flavonoids and phenolic acids is at its highest.

Numerous studies have highlighted a strong correlation between antioxidant activity and total phenolic compound content, particularly in fruits and vegetables. Flavonoids, such as rutin and kaempferol, are particularly potent antioxidant agents capable of neutralising free radicals and protecting against oxidative damage at the cellular level [34]. Additionally, it has been observed that antioxidant capacity tends to decrease in the later stages of ripening, which could be explained by the degradation or transformation of antioxidant metabolites during fruit senescence [23]. The results of this study are somewhat related to those of other authors who have studied the antioxidant activity of different species of *Miconia* [9]

The values obtained in this study are comparable to those reported in previous research on *Miconia crocea*, in which antioxidant activities of 94.7 µmol TE/g DW were recorded using the ABTS method and 33.2 µmol AAE/g DW using the DPPH method [3]. These differences may be due to factors such as extraction methodology, harvest time or genetic variability of the species evaluated.

### 4.4. Antimicrobial Activity

Natural extracts, whose use dates to ancient times to address a wide range of pathologies, from infections to certain types of cancer, remain a significant reserve of therapeutic compounds. Natural products derived from microorganisms, plants and animals constitute a valuable reservoir of bioactive molecules, many of which have served as the basis for the development of new drugs. In the scientific field, the study in question has demonstrated a significant impact on the advancement of disciplines such as synthetic biology and medicinal chemistry. These areas of study have focused on the identification of new antimicrobial entities, which have contributed to the development of innovative treatments and therapeutic approaches. This premise takes on significant relevance in light of the emerging threat posed by antimicrobial resistance at the global level [35].

The presence of a wide range of secondary metabolites in plant structures with antimicrobial potential poses a significant challenge for the efficient extraction of these compounds. In the present study, an experimental design approach has been formulated to optimise the extraction of phenolic compounds, which are recognised for their antimicrobial activity. For this purpose, solvents such as ethanol were used, whose effectiveness in the extraction of bioactive metabolites with antimicrobial properties has been widely documented. In addition, ultrasound-assisted techniques were employed, which have been shown to significantly improve extraction efficiency [36].

The results obtained in this study indicate that the type of solvent is the factor with the most significant influence on the concentration of phenolic compounds extracted. It was observed that a 50% hydroalcoholic solution (ethanol: water) allowed the highest concentrations of total phenols to be achieved. In addition, it was determined that an extraction time of six minutes and three extractions were optimal for maximising the recovery of these compounds. In conclusion, the use of sustainable solvents and efficient extraction techniques is proving to be an imperative in the development of natural antimicrobial agents, not only because of their proven efficacy, but also because of their lower environmental impact and greater compatibility with pharmaceutical and food applications [37].

In this context, the freeze-dried extract of *Miconia crocea* (‘Colcas’) exhibited antimicrobial activity against both Gram-negative and Gram-positive bacterial strains, as well as pathogenic yeasts, at all stages of phenological development. Inhibition was observed against *Escherichia coli*, *Pseudomonas aeruginosa*, *Staphylococcus aureus* and *Streptococcus mutans* in the four maturity stages evaluated. *Candida albicans* was inhibited in all states except M80%, while *C. tropicalis* was only sensitive to extracts from M0% and M100%. These results are consistent with those of studies conducted on different *Miconia* species, in which various extracts have exhibited activity against different microorganisms [9].

The minimum inhibitory concentration (MIC) varied significantly between microorganisms and phenological stages, ranging from 7.8 mg/mL (M0% against *S. mutans*) to 125.0 mg/mL (M100% against *C. tropicalis*). These results suggest that the antimicrobial efficacy of the extract is modulated by the degree of fruit ripeness and the phytochemical composition, being more potent in the early stages of development (M0%), possibly due to the higher concentration of phenolic compounds and flavonoids at these stages.

### 4.5. Haemolytic Activity

The haemolytic activity profile of ‘Colcas’ fruit extract revealed an exceptionally low haemolytic activity across all tested concentrations. The highest concentration evaluated (2500 µg/mL) induced only a minor increase in the weighted optical density, corresponding to a haemolysis rate (%HR) below 3%, which rapidly declined to baseline at 1250 µg/mL and remained negligible at lower doses (Figure 3). This concentration-dependent attenuation confirms the extract’s haemo-compatibility, particularly at physiologically relevant concentrations ≤625 µg/mL, where the %HR was indistinguishable from the negative control. The application of a weighted optical density metric, prioritising the Soret band at 410 nm while incorporating secondary peaks at 540 nm and 580 nm, enhanced the accuracy of the haemolysis quantification by compensating for spectral interferences introduced by the extract’s pigmentation.

The near-absence of haemolytic activity is consistent with the complex mixture of bioactive compounds present in ‘Colcas’ fruit, many of which are recognised for their membrane-protective and antioxidant properties. In particular, the extract’s notable concentrations of ascorbic acid (up to 4.1 mg/100 g DW), anthocyanins (91.3 mg/100 g DW) and phenolic acids—especially chlorogenic acid (4058.8 mg/100 g DW), quercetin and kaempferol—are compounds well documented for their ability to neutralise reactive oxygen species and stabilise cellular membranes [38,39]. These effects are mediated through inhibition of lipid peroxidation and preservation of membrane fluidity, both of which are critical for erythrocyte integrity under oxidative conditions [40,41].

Additional contributors to this protective effect likely include the organic acids malic and citric, which play roles in maintaining intracellular pH and redox balance, as well as carotenoids like lutein (9.1 mg/100 g DW), known to protect against oxidative damage in lipid-rich membranes [42]. The accumulation of pheophytins—chlorophyll degradation products during fruit ripening—may also support erythrocyte protective effects through modulation of redox pathways [43].

Prior studies on comparable botanical extracts corroborate these findings. For instance, extracts from *Amelanchier alnifolia* (Saskatoon berries), which are similarly rich in anthocyanins and phenolic compounds, exhibit <5% haemolytic activity, attributed to their capacity to mitigate oxidative stress in erythrocyte membranes [44]. Likewise, *Nephelium lappaceum* shell extracts—also abundant in chlorogenic acid and quercetin—demonstrated no haemolytic activity [45]. Further supporting evidence comes from studies on other *Miconia* species. The methanolic leaf extract of *Miconia albicans* markedly attenuated haemoglobin release in a clear concentration-dependent manner, demonstrating an antihaemolytic effect consistent with protection of the red cell membrane against oxidative injury [46].

Altogether, the negligible haemolytic activity observed for ‘Colcas’ fruit extract—visualised in Figure 3—can be attributed to a complex synergy of bioactive compounds that collectively reinforce membrane stability and redox resilience. These findings not only support the safety of the extract for potential nutraceutical or pharmaceutical applications but also underscore the value of phytochemical-rich matrices in the design of biocompatible formulations. Future studies in cellular and in vivo models will be essential to delineate the specific mechanisms by which this phytochemical ensemble confers protection to erythrocytes and other cellular systems.

### 4.6. Statistical Analysis

Correlation and principal component analyses of ‘Colcas’ extracts revealed statistically significant associations between bioactive compounds (such as phenols, carotenoids, chlorophylls and organic acids), minerals and antioxidant and antimicrobial activities.

The analysis revealed positive correlations between citric acid and *L*-ascorbic acid, malic acid and *L*-ascorbic acid, lutein and malic acid, and zeaxanthin and *L*-ascorbic acid, as well as between *L*-ascorbic acid and malic acid and lutein. Pheophytin was positively correlated with malic acid, lutein and zeaxanthin, while catechin was positively correlated with chlorophyll b. Furthermore, chlorogenic acid was positively correlated with *L*-ascorbic acid, lutein, zeaxanthin, pheophytin b and syringic acid, while kaempferol was positively correlated with chlorophyll b, catechin and m-coumaric acid. Quercetin was found to be positively correlated with lutein, zeaxanthin, phaeophytin B and chlorogenic acid, as well as quercetin glycoside. Additionally, calcium was found to be positively correlated with 9-*cis* antheraxanthin and violaxanthin, while iron was positively correlated with lutein, 9-*cis* antheraxanthin, chlorogenic acid and quercetin. Potassium was positively correlated with *L*-ascorbic acid, lutein, zeaxanthin, phaeophytin B and chlorogenic acid, while magnesium was positively correlated with 9-*cis* antheraxanthin, violaxanthin and iron. Sodium was positively correlated with chlorophyll b, catechin and kaempferol.

Regarding functional activities, antioxidant activity, as evaluated by the ABTS method, showed a positive correlation with malic acid, lutein, zeaxanthin, phaeophytin a and b, and quercetin. In terms of antimicrobial activity, a positive correlation was observed between activity against *E. coli* and lutein, zeaxanthin, phaeophytin a and b and quercetin; against *P. aeruginosa* and tartaric acid and phaeophytin b; and *S. mutans* with tartaric acid, phaeophytin b, kaempferol and quercetin (ABTS); and against *Candida albicans* and *C. tropicalis* with catechin, *m*-coumaric acid and citric acid.

These correlations are supported by studies indicating a positive relationship between *L*-ascorbic acid, citric acid and malic acid due to their shared metabolic pathways, which are involved in the biosynthesis of primary compounds and the regulation of cellular pH [27]. Similarly, it has been suggested that the biosynthesis of carotenoids is closely linked to that of phenolic compounds, with interaction between these metabolic pathways favouring the simultaneous accumulation of both types of metabolite [47].

Furthermore, the antimicrobial activity of several phenolic compounds, including caffeic, chlorogenic and ferulic acids, against *Staphylococcus aureus* has been documented. Flavonoids such as quercetin have demonstrated vigorous antibiofilm activity against *S. aureus* as well as remarkable antifungal activity against yeasts such as *C. albicans*. Similarly, compounds such as gallic acid, protocatechuic acid and vanillic acid have shown activity against *Salmonella typhimurium* [48]. Previous studies have shown that citric acid inhibits *S. aureus*, *E. coli* and *P. aeruginosa*, while malic acid acts against *E. coli*. Tartaric acid acts against Gram-negative bacteria, such as *E. coli*, and anthocyanins show activity against both *E. coli* and *S. aureus* [6].

As for negative correlations, associations were identified between 9-*cis* anteraxanthine and citric acid; violaxanthine and citric acid; chlorophyll b with tartaric acid; pheophytin b with chlorophyll b; catechin, *m*-coumaric acid and syringic acid with tartaric acid, pheophytin b and gallic acid; kaempferol with tartaric acid, pheophytin b; quercetin glycoside and quercetin with chlorophyll b, catechin, *m*-coumaric acid; calcium with citric acid, magnesium with citric acid; sodium with malic acid, tartaric acid, lutein, zeaxanthin, phaeophytin a, phaeophytin b, quercetin glycoside and quercetin; antioxidant activity by ABTS and DPPH and antimicrobial activity against *E. coli*, *S. aureus*, *P aeruginosa* and *S. mutans* with chlorophyll b, kaempferol and sodium; antimicrobial activity against *C. albicans* with tartaric acid, 9-*cis* antheraxanthine; antimicrobial activity against *C. tropicalis* with 9-*cis*-antheraxanthine, violaxanthine, quercetin glucoside, quercetin, calcium, iron and magnesium.

Conversely, principal component analysis revealed that component 1 accounted for 47.5% of the total variability, while component 2 accounted for 23.7%, resulting in a cumulative contribution of 71.2%. The variables that contributed most to the overall variability were phaeophytin b, quercetin, zeaxanthin, lutein, potassium, *L*-ascorbic acid and antimicrobial activity against *Candida albicans*. Antioxidant activity against the ABTS radical was primarily influenced by quercetin, phaeophytin b, phaeophytin a, zeaxanthin and lutein. In contrast, antioxidant activity measured by DPPH was primarily associated with chlorophyll B, kaempferol and *p*-coumaric acid. In contrast, antimicrobial activity against *E. coli*, *S. aureus* and *P. aeruginosa* was more strongly associated with malic acid, lutein, phaeophytin a, zeaxanthin, quercetin, phaeophytin b, quercetin glycoside and violaxanthin. For *C. albicans*, the most influential components were syringic acid and *m*-coumaric acid. For *C. tropicalis*, *m*-coumaric acid, chlorophyll b and kaempferol were the most influential.

## 5. Conclusions

The *Miconia* genus has been used in traditional medicine, but little information is currently available on *Miconia crocea* (‘Colcas’). The results showed that, as the fruit ripened, the pH decreased and the levels of soluble solids and titratable acidity increased, which is consistent with the metabolic changes that are typical of fruit development. Potassium was the predominant mineral. Malic acid was the main organic acid. Lutein was the most abundant carotenoid. Total anthocyanin content increased significantly towards maturity, and *p*-coumaric acid and chlorogenic acid were the most abundant phenolic compounds. Antioxidant activity, as assessed by the ABTS and DPPH methods, showed agreement. The optimal extraction process for phenolic compounds consisted of 50% ethanol, six minutes of ultrasound and three consecutive extractions. In antimicrobial evaluations, the M0% extract exhibited the lowest minimum inhibitory concentration against *S. mutans* and demonstrated inhibitory activity against *E. coli*, *P. aeruginosa*, *S. aureus*, *C. albicans* and *C. tropicalis*. In addition, antihaemolytic activity confirmed biocompatibility. From a biological perspective, these variations could be related to the species’ adaptation mechanisms in response to environmental conditions. From a technological perspective, they provide helpful information on how to utilise the fruit at various stages of ripeness, depending on the compound of interest. It is important to note, however, that the tests performed were exclusively in vitro (ABTS, DPPH, MIC and antihaemolytic), which limits the direct extrapolation of the results to in vivo conditions. Similarly, the data refer to a specific population of fruits and may therefore vary depending on agroecological and environmental factors. Further studies are therefore needed to investigate the toxicity and stability of these compounds, as well as the technological feasibility of incorporating them into food or therapeutic matrices. The results obtained provide a solid foundation for future research aimed at validating the safety and efficacy of this underutilised Andean species, as well as exploring its responsible use.

## Figures and Tables

**Table 1:** Average values of the physicochemical characteristics of ‘Colcas’ at different phenological stages.

Parameters *	M0%			M50%			M80%			M100%		
pH	3.4	±	0.1 ^a^	3.2	±	0.0 ^b^	3.0	±	0.0 ^c^	2.8	±	0.0 ^d^
SS (°Brix)	1.2	±	0.3 ^c^	1.1	±	0.1 ^c^	7.0	±	0.0 ^a^	6.2	±	0.3 ^b^
TA (%)	0.3	±	0.1 ^d^	0.6	±	0.1 ^c^	0.8	±	0.2 ^b^	1.2	±	0.1 ^a^
Humidity (%)	81.5	±	0.5 ^b^	86.5	±	0.7 ^a^	86.0	±	1.2 ^a^	87.2	±	1.3 ^a^
Ash (%)	1.0	±	0.2 ^b^	1.2	±	0.3 ^ab^	1.4	±	0.1 ^a^	0.8	±	0.1 ^c^
Mineral profile (mg/100 g DW) **												
Ca	1263.9	±	11.7 ^b^	93.0	±	4.0 ^c^	2760.8	±	24.1 ^a^	473.7	±	64.1 ^c^
Fe	nd			8.6	±	0.4 ^b^	22.0	±	0.4 ^a^	nd		
K	1154.6	±	10.3 ^c^	3066.7	±	22.7 ^a^	3109.5	±	11.3 ^a^	1728.0	±	23.9 ^b^
Mg	141.8	±	7.7 ^c^	41.7	±	0.5 ^c^	254.4	±	58.8 ^a^	106.8	±	17.6 ^b^
Na	24.9	±	6.9 ^a^	5.8	±	0.2 ^c^	9.0	±	1.5 ^b^	8.0	±	0.9 ^b^

Note: The values are means ± standard deviation (*, N° samples = 20 and **, N° samples = 3); SS, soluble solids; TA, titratable total acidity; nd, undetectable limit. Different lowercase letters indicate significant differences between stages of ripeness.

**Table 2:** Average values of bioactive compounds of ‘Colcas’ at different phenological stages.

Parameters	M0%			M50%			M80%			M100%		
Vitamin C (mg/100 g DW)	2.0	±	0.0 ^b^	4.1	±	0.4 ^a^	2.2	±	0.2 ^b^	0.9	±	0.0 ^c^
Organic acid profile (mg/100 g DW)												
Citric acid	387.5	±	32.2 ^c^	1059.8	±	67.0 ^a^	128.1	±	5.0 ^d^	581.9	±	27.0 ^b^
Malic acid	3722.5	±	20.7 ^d^	5280.5	±	37.7 ^a^	4580.9	±	38.2 ^b^	4099.8	±	54.3 ^c^
Tartaric acid	62.1	±	6.6 ^d^	366.3	±	26.0 ^c^	659.5	±	98.8 ^b^	730.6	±	205.3 ^a^
Total organic acid	4172.1	±	6.6 ^c^	6706.6	±	26.0 ^abc^	5368.5	±	98.8 ^ab^	5421.2	±	205.3 ^a^
Carotenoid profile (mg/100 g DW)												
Lutein	2.9	±	1.0 ^d^	9.1	±	0.7 ^b^	6.9	±	0.0 ^b^	4.4	±	1.0 ^c^
9-*cis*-Anteraxanthin	0.2	±	0.0 ^c^	0.2	±	0.0 ^c^	0.6	±	0.0 ^a^	0.3	±	0.0 ^b^
Violaxanthin	nd			0.2	±	0.0 ^b^	2.1	±	0.2 ^a^	nd		
Zeaxanthin	nd			0.5	±	0.1 ^a^	0.3	±	0.0 ^b^	0.1	±	0.0 ^c^
Total carotenoid	3.1	±	1.0 ^c^	10.0	±	0.8 ^a^	9.9	±	0.3 ^a^	4.8	±	1.1 ^b^
Chlorophylls and their derivatives (mg/100 g DW)												
Chlorophyll b	15.0	±	0.1 ^b^	3.0	±	0.2 ^a^	1.7	±	0.2 ^b^	0.1	±	0.0 ^c^
Pheophytin a	4.3	±	0.5 ^c^	10.3	±	1.7 ^a^	8.0	±	1.1 ^a^	6.3	±	0.6 ^b^
Pheophytin b	28.0	±	2.3 ^d^	88.7	±	4.9 ^a^	79.5	±	0.4 ^b^	70.7	±	3.8 ^c^
Total chlorophyll	47.3	±	0.3 ^d^	102.0	±	0.7 ^a^	89.2	±	3.2 ^b^	77.1	±	2.4 ^c^
Total anthocyanins (mg/100 g DW)	10.9	±	1.2 ^d^	52.7	±	2.9 ^c^	75.2	±	6.3 ^b^	91.3	±	5.5 ^a^
Phenolic compounds (mg/100 g DW)												
Gallic acid	0.02	±	0.0 ^b^	0.02	±	0.0 ^b^	0.02	±	0.0 ^b^	0.03	±	0.0 ^a^
Catechin	433.3	±	25.5	229.8	±	25.6	210.9	±	30.7	93.6	±	5.4
*m*-Cumaric acid	3308.1	±	59.2 ^a^	2239.2	±	33.7 ^b^	1551.1	±	35.3 ^c^	735.8	±	10.5 ^d^
Syringic acid	544.1	±	15.5 ^a^	628.2	±	10.1 ^b^	508.7	±	96.6 ^c^	211.5	±	9.8 ^d^
Chlorogenic acid	2303.8	±	22.6 ^c^	3996.9	±	291.5 ^b^	4058.8	±	179.8 ^a^	1402.2	±	338.5 ^d^
Kaempferol	1636.0	±	21.6 ^a^	342.4	±	7.4 ^b^	456.8	±	1.0 ^b^	348.2	±	8.5 ^c^
Quercetin glucoside	183.4	±	5.4 ^c^	272.2	±	15.1 ^b^	307.7	±	40.3 ^a^	280.4	±	40.3 ^b^
Quercetin	36.9	±	3.4 ^c^	88.5	±	7.1 ^a^	99.0	±	3.7 ^a^	62.8	±	4.5 ^b^
Total phenolics	7145.6	±	581.0 ^b^	7539.9	±	39.1 ^a^	6835.7	±	70.2 ^c^	2844.5	±	51.3 ^d^

Note: The values are means ± standard deviation (n = 3). nd, undetectable limit. Different lowercase letters indicate significant differences between stages of ripeness.

**Table 3:** Average values of antioxidant activity of ‘Colcas’ at different phenological stages.

	M0%			M50%			M80%			M100%		
Antioxidant activity (mmol TE/100 g DW)												
DPPH	5.5	±	0.1 ^a^	5.1	±	0.2 ^a^	5.2	±	0.1 ^a^	5.3	±	0.1 ^a^
ABTS	5.5	±	0.11 ^b^	6.1	±	0.21 ^a^	6.1	±	0.02 ^a^	5.8	±	0.15 ^b^

Note: The values are means ± standard deviation (n = 3). Different lowercase letters indicate significant differences between stages of ripeness.

**Table 4:** Average values of the antimicrobial activity of ‘Colcas’ at different phenological stages.

Microorganisms	Inhibition Zone (mm)											
	M0%			M50%			M80%			M100%		
*E. coli* ATCC 8739	13.5	±	0.7 ^b^	16.0	±	2.8 ^a^	18.5	±	3.5 ^a^	18.0	±	3.0 ^a^
*P. aeruginosa* ATCC 9027	14.0	±	0.0 ^c^	19.0	±	1.4 ^b^	19.5	±	3.5 ^b^	26.0	±	0.0 ^a^
*S. aureus* ATCC 6538P	18.0	±	2.8 ^b^	23.5	±	2.1 ^a^	23.5	±	2.1 ^a^	24.5	±	0.0 ^a^
*S. mutans* ATCC 25175	17.5	±	0.7 ^b^	24.0	±	1.4 ^a^	24.5	±	0.7 ^a^	25.0	±	2.8 ^a^
*C. albicans* ATCC 1031	17.0	±	0.0 ^a^	15.0	±	0.0 ^b^	-			8.0	±	1.0 ^c^
*C. tropicalis* ATCC 13803	13.0	±	1.4 ^a^	-			-			10.0	±	0.0 ^b^

Note: The values are means ± standard deviation (n = 3); -: No zone at concentration tested. Different lowercase letters indicate significant differences between stages of ripeness.

**Table 5:** Minimal inhibitory concentration of ‘Colcas’ at different phenological stages.

Microorganisms	Minimal Inhibitory Concentration (mg/mL)	
	M0%	M100%
*E. coli* ATCC 8739	62.5	62.5
*P. aeruginosa* ATCC 9027	31.3	31.3
*S. aureus* ATCC 6538P	15.6	15.6
*S. mutans* ATCC 25175	7.8	15.6
*C. albicans* ATCC 1031	31.3	62.5
*C. tropicalis* ATCC 13803	62.5	125.0

Note: The values are means ± standard deviation (n = 3).

## Data Availability

The original contributions presented in this study are included in the article. Further inquiries can be directed to the corresponding author.
